# Targeted Deletion of Thymosin Beta 4 in Hepatic Stellate Cells Ameliorates Liver Fibrosis in a Transgenic Mouse Model

**DOI:** 10.3390/cells12121658

**Published:** 2023-06-18

**Authors:** Jieun Kim, Chanbin Lee, Jinsol Han, Hayeong Jeong, Sihyung Wang, Yung Hyun Choi, Youngmi Jung

**Affiliations:** 1 Institute of System Biology, Pusan National University, Pusan 46241, Republic of Korea; jieun@pusan.ac.kr (J.K.); chanbin@pusan.ac.kr (C.L.); 2 Department of Integrated Biological Science, College of Natural Sciences, Pusan National University, Pusan 46241, Republic of Korea; wlsthf1408@pusan.ac.kr (J.H.); jeong17@pusan.ac.kr (H.J.); 3 New Drug Development Center, Daegu Gyeongbuk Medical Innovation Foundation, Daegu 41061, Republic of Korea; shwang@kmedihub.re.kr; 4 Department of Biochemistry, Dong-Eui University College of Korean Medicine, Pusan 47227, Republic of Korea; choiyh@deu.ac.kr; 5 Department of Biological Sciences, College of Natural Sciences, Pusan National University, Pusan 46241, Republic of Korea

**Keywords:** Tβ4, Tβ4-flox transgenic mice, hepatic stellate cells, liver fibrosis

## Abstract

Liver fibrosis is the most common feature of liver disease, and activated hepatic stellate cells (HSCs) are the main contributors to liver fibrosis. Thus, finding key targets that modulate HSC activation is important to prevent liver fibrosis. Previously, we showed that thymosin β4 (Tβ4) influenced HSC activation by interacting with the Hedgehog pathway in vitro. Herein, we generated Tβ4 conditional knockout (Tβ4-flox) mice to investigate in vivo functions of Tβ4 in liver fibrosis. To selectively delete Tβ4 in activated HSCs, double-transgenic (DTG) mice were generated by mating Tβ4-flox mice with α-smooth muscle actin (α-Sma)-Cre-ER^T2^ mice, and these mice were administered carbon tetrachloride (CCl_4_) or underwent bile duct ligation to induce liver fibrosis. Tβ4 was selectively suppressed in the activated HSCs of DTG mouse liver, and this reduction attenuated liver injury, including fibrosis, in both fibrotic models by repressing Hedgehog (Hh) signaling. In addition, the re-expression of Tβ4 by an adeno-associated virus reversed the effect of HSC-specific Tβ4 deletion and led to liver fibrosis with Hh activation in CCl_4_-exposed mice treated with tamoxifen. In conclusion, our results demonstrate that Tβ4 is a crucial regulator of HSC activation, suggesting it as a novel therapeutic target for curing liver fibrosis.

## 1. Introduction

Liver fibrosis, which is scarring of the liver, is the most common consequence of liver injuries, including excessive fat accumulation, viral infection, alcohol consumption, and cholestasis [1]. The activation of hepatic stellate cells (HSCs) is the substantial event in the development and progression of liver fibrosis, and activated/myofibroblastic HSCs are mainly responsible for the synthesis and infiltration of the extracellular matrix (ECM), such as collagens [1,2,3]. Thus, controlling HSC activation is considered an important strategy to alleviate chronic liver disease, including liver fibrosis; however, the key factors that target and control HSC activation remain to be fully elucidated.

Thymosin beta 4 (Tβ4), a naturally occurring peptide containing 43 amino acids, is a member of the highly conserved thymosin beta family [4,5]. This endogenous peptide is produced in higher concentrations where tissues are damaged and modulates multiple cellular activities, including cell motility, survival, wound healing, and inflammation [4,6,7,8]. Tβ4 is regarded as a multifunctional regenerative peptide, and many researchers and companies have attempted to develop a synthetic copy of Tβ4 as a novel regenerative medicine to treat various diseases, such as dermal wounds, corneal injuries, cardiovascular disease, and neurological disorders [4,9]. In experimental animal studies, exogenous Tβ4 administration has been shown to have beneficial effects on myocardial infarction, stroke, hair loss, and dry eye [4,9,10,11,12]. There have also been several clinical trials assessing synthetic Tβ4 treatment in dry eye syndrome and neurotrophic keratitis [13,14,15]. However, there is still a huge limitation in the application of Tβ4 as a treatment because many studies have only focused on the visible effects of exogenous or synthetic Tβ4, but not endogenous Tβ4, and have overlooked the underlying importance of endogenous Tβ4 under physiological and pathophysiological conditions [5]. Therefore, the functions of Tβ4 produced but not supplemented in the body remain unknown.

Accumulating evidence suggests that Tβ4 is involved in the development of hepatic fibrosis and is specifically associated with HSC activation [5,16,17]. As with Tβ4 studies in other organs, most experimental studies have focused on the actions of exogenous Tβ4 on liver fibrosis and reported that exogenous Tβ4 could attenuate liver fibrosis by modulating HSC activation [5,18,19,20]. However, until recently, researchers have been interested in the roles of endogenous Tβ4 in the liver. Our previous studies revealed that Tβ4 is expressed by activated HSCs in the chronically injured liver and impacts HSC activation and liver fibrosis by directly interacting with Hedgehog (Hh) signaling, which is a main controller of HSC activation and fibrosis [16,17]. In vitro suppression of Tβ4 decreased the expression of smoothened (Smo), an effector of the Hh pathway, and GLI family zinc finger 2 (Gli2), a transcription factor in the Hh pathway, and it inactivated HSCs, suggesting that Tβ4 was a potential regulator of HSC activation [16]. However, these findings were obtained from in vitro experiments, and it remains a significant challenge to investigate the in vivo effect of Tβ4 on liver fibrosis in the context of Tβ4 suppression because of the absence of available Tβ4-knockout mice.

In the current study, we created Tβ4 conditional knockout (Tβ4-flox) mice harboring floxed Tβ4 alleles by using the Cre-LoxP system. To induce activated HSC-targeted Tβ4 knockout, double-transgenic (DTG) mice were obtained by mating Tβ4-flox mice with α-smooth muscle actin (α-Sma)-Cre-ER^T2^ mice with tamoxifen-inducible Cre activity under the control of the α-Sma promoter [21,22]. α-Sma is a marker of activated HSCs [3,22]. Based on our previous findings showing that Tβ4 was primarily expressed by activated HSCs and that Tβ4 suppression in HSCs inhibited HSC activation [16,17], we investigated the physiological function of activated HSC-specific deletion of Tβ4 in the experimental liver fibrosis models. Herein, we demonstrate that HSC-targeted Tβ4 depletion attenuates the activation of HSCs and hepatic fibrosis, suggesting Tβ4 as an attractive target to develop therapeutic agents for liver fibrosis and that Tβ4-flox mice are a useful experimental model for use in future studies to further understand the functions of endogenous Tβ4 in other diseases and organs.

## 2. Materials and Methods

### 2.1. Generation of Tβ4-Floxed Mice

The conditional knock-out Tβ4 mice were generated by Macrogen, Inc. using CRISPR/Cas9 system and were maintained in pathogen-free condition at Macrogen (Seoul, Republic of Korea). Cas9 nuclease (M0386; New England Biolabs Inc., Ipswich, MA, USA) and guide RNAs (gRNAs) to target intron 1–2 and intron 2–3 of Tβ4 gene were prepared by Macrogen using GeneArt™ Precision gRNA Synthesis Kit (A29377, Thermo Fisher Scientific, Waltham, MA, USA). Double-stranded donor DNA (dsDonor) was synthesized from Invitrogen. Two sets of gRNAs (gRNA#1: ACG AAG GTG GAA ATA GTC AAA GG, gRNA#2: TTA GCA TGT CTT GCT TAG CGA GG) were synthesized and validated activity using in vitro cleavage reactions. Briefly, amplified Tβ4 gene as template was incubated for 90 min at 37 °C with Cas9 nuclease (20 nm) and gRNA (40 nm) in 1× NEB 3 buffer. Reactions were terminated with 6× stop solution containing 30% glycerol, 1.2% SDS, and 100 mM EDTA. Mixtures were performed electrophoresis and confirmed cleavage activity. After confirming in vitro cleavage reactions, microinjection of CRISPR/Cas9 was performed. Briefly, pregnant mare serum gonadotropin (PMSG) and human chorionic gonadotropin (hCG) were treated into C57BL/6N female mice. After 48 h, these female mice were mated with C57BL/6N stud male mice. Next day, female mice showing vaginal plug were sacrificed, and fertilized embryos are harvested. The mixture of Cas9 nuclease, two sets of sgRNAs, and dsDonor were microinjected into one cell embryo. Microinjected embryos were incubated at 37 °C for 2 h and then microinjected embryos at the 14-to-16-cell stage were transplanted into oviducts of pseudo-pregnant recipient mice. After F0 founders were born, genotyping for loxP insertion was performed with a set of 2 primers (Forward primer: GGG GGA TGC TAA ACT CTT CC; Reverse primer: ACC CAG CTT TTT CCT TCA CC) and the expected PCR fragment size is 1802-bp. Then, the fragment was digested with AflIII restriction enzyme to confirm the positive sample. Enzyme-digested positive samples were performed via TA cloning and analyzed by Sequencing. After breeding of F0 founders by Macrogen, we obtained the Tβ4^flox/+^ offspring from Macrogen (Seoul, Republic of Korea) and maintained these mice over generations.

### 2.2. Animal Experiments

Male C57BL/6 mice (6-week-old mice) were purchased from Hyochang (Daegu, Republic of Korea) and used for wild-type (WT) controls. All mice were provided an adequate acclimation period to allow them to stabilize to their new environment. The α-Sma-Cre-ER^T2^ transgenic mice, which are originally made by Dr. Pierre Chambon (IGBMC), were kindly provided by Dr. Anna Mae Diehl (Duke University Medical Center) [21,22]. Cre-positive and homozygous Tβ4^flox/flox^ DTG mice were bred by crossing α-Sma-Cre-ER^T2^ hemizygous, Tβ4^flox/flox^ homozygous mice with Tβ4^flox/flox^ homozygous mice (Tβ4-flox mice). Genotyping for α-Sma-Cre-ER^T2^ was performed using a set of 4 primers (PB003: TGC AAC GAG TGA GGT TCG C; PB004: GAT CCT GGC AAT TTC GGC TAT ACG; 1136: GGT TTC TAT TGC TAC CAA GAG ACA T; 1044: TGC ACC AAA CCC TGG ACT AAG CAT). Genotyping for the floxed Tβ4 allele was performed using a set of 2 primers (Forward primer: GGA AAT GGC TTC GAT CTA TCC; Reverse primer: CGA GTC CTC TCT AAA GAT CTT CC). After genotyping, each newly weaned DTG mice were randomly introduced in a clean cage. All mice were housed in groups (maximum of 5 mice per cage) in ventilated, temperature- and humidity-controlled cages with an absorbent bedding material under a 12 h light/12 h dark cycle with free access to standard food and water. All animals were treated the same way, and the time of treatment was always the same.

For the induction of toxic liver fibrosis, 7-week-old C57BL/6 WT or DTG male mice were treated with four intraperitoneal injections of 100 μL of CCl_4_ (0.5 μL per g body weight, dissolved in corn oil) or corn oil (CON) (*n* = 4–5 mice per group), as described previously [23]. Animals were sacrificed at 2 days after the last CCl_4_ injection. For the induction of cholestatic liver fibrosis, 8-week-old DTG male mice were subjected to either BDL or sham surgery under isoflurane anesthesia (*n* = 4–5 mice per group), as described previously [22]. Animals were sacrificed at 3 weeks after surgery. To initiate Cre-mediate gene rearrangement of floxed allele in CCl_4_ model, mice were injected intraperitoneally with tamoxifen (TMX, Sigma-Aldrich, St. Louis, MO, USA) dissolved in corn oil. In CCl_4_ model, α-Sma-Cre-ER^T2^ activity was induced by 6 intraperitoneal injections of TMX (2 mg per mice) starting 3 days after the first CCl_4_ injection and ending one day before the last CCl_4_ injection as previously described [23]: CON + VEH (*n* = 4), CON + TMX (*n* = 4), CCl_4_ + VEH (*n* = 5), and CCl_4_ + TMX (*n* = 5). In BDL model, TMX (10 mg per kg body weight) was delivered every other day (total of 9 intraperitoneal injections) starting at 4 days after the BDL surgery until 3 weeks after surgery as previously described [22]: Sham + VEH (*n* = 4), Sham + TMX (*n* = 4), BDL + VEH (*n* = 5), and BDL + TMX (*n* = 5). To assess the effect of Tβ4 re-expression in the livers of TMX-treated DTG mice, AAV6 vectors containing a Tβ4 gene were used to conditionally enhance the Tβ4 expression in the liver, mainly HSCs [24]. Under the identical CCl_4_ and TMX regimen, DTG mice were intravenously injected via tail vein with 5 × 10^10^ viral genome copies of AAV6-CMV-Con or AAV6-CMV-Tβ4 (Vector Biolabs) after 3rd injection of CCl_4_, and then they received residual TMX and Tβ4 injections: CON + VEH + AAV6-Con, CCl_4_ + VEH + AAV6-Con, CCl_4_ + TMX + AAV6-Con, and CCl_4_ + TMX + AAV6-Tβ4 (*n* = 4 per group). Vector injections were performed slowly, and the volume was limited to 100μL for tail vein injection. At the end of each time point, mice were sacrificed to collect blood and liver samples. Animal care and surgical procedures were approved by the Pusan National University–Institutional Animal Care and Use Committee and carried out in accordance with the provisions of the National Institutes of the National Institutes of Health Guide for the Care and Use of Laboratory Animals (Approval Number PNU-2016-1286 and PNU-2020-2652).

### 2.3. RNA Analysis

Total RNA was extracted from pHSCs or liver tissues using Trizol reagent (Invitrogen, Carlsbad, CA, USA). The concentration and purity of RNA were determined using nanodrop. Template complementary DNA was synthesized from total RNA using the SuperScript II First-strand synthesis system (Invitrogen, Carlsbad, CA, USA) according to the manufacturer’s protocols. Real-time qRT-PCR analysis was performed on QuantStudio 1 (Thermo Scientific, Pleasanton, CA, USA) using POWER SYBR Green Master Mix (Applied Biosystems, Foster City, CA, USA) on the manufacturer’s instructions. All reactions were duplicated, and data were analyzed according to the ΔΔCt method. The expression values were normalized to the levels of mouse 40S ribosomal protein S9 mRNA. The sequences of all primers used in this study are summarized in Table S1. All PCR products were directly sequenced for genetic confirmation (Macrogen, Inc., Seoul, Republic of Korea).

### 2.4. Western Blot Assay

Total protein was extracted from pHSCs or freeze-clamped liver tissues that had been stored at −80 °C. Samples were homogenized in triton lysis buffer supplemented with protease inhibitors (Complete mini; Roche, Indianapolis, IN, USA) and centrifuged at 13,000 r.c.f. for 15 min. The supernatants containing protein extracts were used in subsequent biochemical analysis. Protein concentration was measured by Pierce BCA Protein Assay Kit (Thermo Fisher Scientific, Waltham, MA, USA). Equal amount of total protein lysates was separated by SDS–polyacrylamide gel electrophoresis (PAGE) and then transferred onto a polyvinylidene difluoride (PVDF) membrane (Millipore, Darmstadt, Germany). Primary antibodies used in this study were as follows: rabbit anti-Tβ4 antibody (A9520; diluted 1:5000; Immundiagnostik AG, Bensheim, Germany), rabbit anti-Smo antibody (ab72130; diluted 1:1000; Abcam, Cambridge, MA, USA), rabbit anti-Gli2 antibody (GWB-CE7858; diluted 1:1000; GenWay Biotech, Inc., San Diego, CA, USA), rabbit anti-pGSK-3α/β antibody (9331; diluted 1:1000; Cell Signaling Technology, Inc., Danvers, MA, USA), rabbit anti-GSK-3β antibody (9315; diluted 1:1000; Cell Signaling Technology, Inc., Danvers, MA, USA), rabbit anti-TGF-β antibody (3711S; diluted 1:1000; Cell Signaling Technology, Inc., Danvers, MA, USA), mouse anti-Cre Recombinase antibody (MAB3120; diluted 1:500; Sigma-Aldrich, St. Louis, MO, USA), mouse anti-ILK antibody (611802; diluted 1:1000; BD Biosciences, San Diego, CA, USA), mouse anti-α-Sma antibody (A5228; diluted 1:1000; Sigma-Aldrich, St. Louis, MO, USA), and mouse anti-GAPDH antibody (MCA4739; diluted 1:1000; AbD Serotec, Oxford, UK). Horseradish peroxidase-conjugated anti-rabbit or anti-mouse IgG (Enzo Life Sciences, Inc., Farmingdale, NY, USA) was used as secondary antibody. Protein bands were detected using an EzWestLumi ECL solution (ATTO Corporation, Tokyo, Japan) as per the manufacturer’s specifications (ATTO Corporation, Ez-Capture II, Tokyo, Japan,). Densities of protein bands were measured using CS Analyzer software (Version 3.00.1011, ATTO & Rise Corporation, Tokyo, Japan,).

### 2.5. Liver Histology and Immunohistochemistry

Liver specimens were fixed in 10% neutral buffered formalin, embedded in paraffin, and cut into 4 μm sections and placed on glass slides. To examine hepatic morphology and assess liver fibrosis, liver sections were deparaffinized, rehydrated, and stained via the usual method with standard hematoxylin and eosin staining (H&E) and Sirius red staining as previously described [16,25]. For immunohistochemical staining, liver sections were deparaffinized, rehydrated, and then incubated for 10 min in 3% hydrogen peroxide to block endogenous peroxidase. Antigen retrieval was performed by heating in 10 mM sodium citrate buffer (pH 6.0). Sections were blocked in DAKO protein block (X9090; DAKO, Carpinteria, CA, USA) for 30 min and incubated with primary antibodies at 4 °C overnight. The following primary antibodies were used: Tβ4 (A9520; diluted 1:8000; Immundiagnostik AG, Bensheim, Germany) and α-Sma (ab5694; diluted 1:1000; Abcam, Cambridge, MA, USA). Polymer horseradish peroxidase (HRP)-conjugated anti-rabbit secondary antibody (K4003; DAKO, Carpinteria, CA, USA) was used as secondary antibody. 3,3′-Diaminobenzidine (DAB) (K3466; DAKO, Carpinteria, CA, USA) was employed for the detection procedure.

### 2.6. HSC Isolation and Culture

Primary HSCs were isolated as described previously [16,25]. Briefly, mice were anaesthetized with isoflurane to immobilize in the recumbent position on a treatment table, and the inferior vena cava was cannulated under aseptic conditions. Livers were perfused in situ with EGTA and collagenase (Sigma-Aldrich, St. Louis, MO, USA), to disperse the cells. Primary HSCs were separated by differential centrifugation on OptiPrep (Sigma-Aldrich, St. Louis, MO, USA) density gradient and located on the upper layer of 11.5% OptiPrep. The purity of HSCs was >98%, as established by microscopy examination for lipid droplets and vitamin A auto-fluorescence. Isolated HSCs were seeded at a density of 3 × 10^2^ cells/mm^2^ and cultured in DMEM medium (Gibco, Thermo Fisher Scientific, Waltham, MA, USA) supplemented with 10% FBS and 1% P/S at 37 °C in a humidified atmosphere containing 5% CO_2_.

### 2.7. Adenoviral Transfection in Primary HSCs Isolated from Mice

HSCs isolated from Tβ4-flox male mice were cultured for 3 days, and then starved in serum-depleted medium for overnight. Adenoviruses harboring either the GFP gene (AdGFP, Vector Biolabs, Malvern, PA, USA) or Cre recombinase gene (AdCre, Vector Biolabs, Malvern, PA, USA) were added to these pHSCs at a multiplicity of infection (MOI) of 80, as described previously [16]. After 24 h, virus-containing medium was aspirated and replaced with fresh medium. Viral efficiency of AdGFP infection was assessed by fluorescent microscope, with >95% of infected cells found to be GFP positive.

### 2.8. Measurement of ALT and AST

Serum aspartate aminotransferase (AST/GOT, glutamate-oxaloacetate transaminase) and alanine aminotransferase (ALT/GPT, glutamate pyruvate transaminase) were measured using GOT reagents (AM103-K; Asan Pharmaceutical, Seoul, Republic of Korea) and GPT reagents (AM102-K; Asan Pharmaceutical, Seoul, Republic of Korea) according to the manufacturer’s instructions.

### 2.9. Hydroxyproline Assay

Hydroxyproline content of the livers was calculated by the method previously described [16,25]. Briefly, 50 mg of freeze liver tissue was hydrolyzed in 6N HCl at 110 °C for 16 h. The hydrolysate was evaporated under vacuum and the sediment was re-dissolved in 1 mL distilled water. Sample were filtered using 0.22 μm filter centrifuge tube (Corning Incorporated) at 14,000 r.p.m. for 5 min. Then, 0.5 mL of chloramine-T solution, containing 1.41 g of chloramine-T dissolved in 80 mL of acetate–citrate buffer and 20 mL of 50% isopropanol, was added and incubated at room temperature for 20 min. Then, 0.5 mL of Ehrlich’s solution, containing 7.5 g of dimethylaminobenzaldehyde dissolved in 13 mL of 60% perchloric acid and 30 mL of isopropanol, was added to the mixture and incubated at 65 °C for 15 min. After cooling at room temperature, the standard and samples were measured by a spectrophotometer at 561 nm. Amount of hydroxyproline in each sample was determined using regression curve from high purity Trans-4-hydroxy-L-proline (Sigma-Aldrich, St. Louis, MO, USA) as a standard. Total hydroxyproline was calculated based on individual liver weights (mg hydroxyproline/mg liver).

### 2.10. Statistics

Results are expressed as mean ± s.e.m. Statistical analysis was analyzed by two-tailed unpaired Student’s *t*-test. Differences were considered as statistically significant when *p*-values < 0.05. Statistical analyses were performed using Microsoft Excel and GraphPad Prism 8 (GraphPad Software Inc., Boston, MA, USA).

## 3. Results

### 3.1. Generation of Tβ4 Conditional Knockout Mice

**Figure 1:**
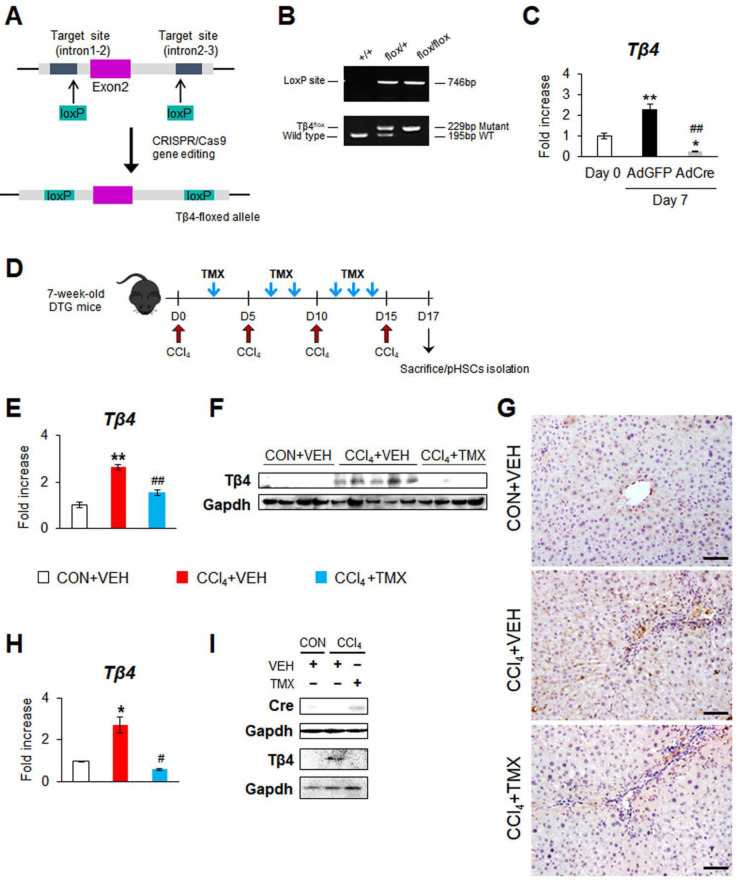
Generation of the Tβ4-floxed mice and tamoxifen-induced Tβ4 deletion in CCl_4_-injected DTG mice and primary HSCs isolated from these mice. (**A**) Schematic illustration to generate a conditional allele at the Tβ4 locus. Intron 1–2 and Intron 2–3 of Tβ4 gene was targeted by two guide RNAs that were designed to cut both ends of the exon 2 of Tβ4. By CRISPR/Cas9 gene editing tool, two LoxP sequences are inserted at both ends of the exon 2 by single-stranded donor oligonucleotides, generating the Tβ4-floxed allele having target exon 2 with flanking loxP sites. (**B**) PCR-based genotyping for the LoxP site and the floxed Tβ4 alleles. Genomic DNA extracted from mice were amplified using specific primers. Wild-type Tβ4^+/+^, hemizygous Tβ4^flox/+^, and homozygous Tβ4^flox/flox^ (Tβ4-flox) mice were clearly distinguished by detecting the presence of LoxP site (746-bp size in product) and the floxed Tβ4 allele (WT: 195-bp, mutant: 229-bp size in product). (**C**) qRT-PCR analysis of Tβ4 in primary mouse HSCs (pHSCs) at Day 0 and AdGFP or AdCre-transfected pHSCs at Day 7. These cells were isolated from livers of Tβ4-flox mice. Results are normalized to pHSCs at Day 0. Results of relative expression values are graphed as mean ± s.e.m. of triplicate experiments (Student’s *t*-test; * *p* < 0.05, ** *p* < 0.005 vs. Day0, ^##^ *p* < 0.005 vs. AdGFP) (**D**) Schematic diagram showing the timing of carbon tetrachloride (CCl_4_) and tamoxifen (TMX) injection into double-transgenic (DTG) mice. At 2 days after the last injection of CCl_4_, mice were sacrificed to harvest tissue samples or isolate primary HSC (pHSC). (**E**) qRT-PCR analysis of hepatic Tβ4 in representative DTG mice receiving corn oil (CON) + vehicle (VEH) (*n* = 4), CCl_4_ + VEH (*n* = 5), or CCl_4_ + TMX (*n* = 4). (**F**) Representative immunoblots of hepatic Tβ4 in representative mice from each group, CON + VEH (*n* = 4), CCl_4_ + VEH (*n* = 5), and CCl_4_ + TMX (*n* = 4). Gapdh was used as an internal control. (**G**) Representative images of immunostaining for Tβ4 in liver sections from each group (scale bar = 50 μm). (**H**) qRT-PCR analysis of Tβ4 in pHSCs isolated from livers of representative mice from each group (*n* = 3 per group). Results of relative expression values are graphed as mean ± s.e.m. (Student’s *t*-test; * *p* < 0.05, ** *p* < 0.005 vs. CON + VEH group, ^#^ *p* < 0.05, ^##^ *p* < 0.005 vs. CCl_4_ + VEH group). (**I**) Immunoblots of Cre and Tβ4 in pHSCs isolated from livers of mice from each group. Gapdh was used as an internal control. Immunoblots shown represent one of three experiments with similar results.

To elucidate the precise function of Tβ4 in vivo, we generated Tβ4 conditional knockout mice harboring the floxed Tβ4 allele by CRISPR/Cas9-mediated gene editing (Figure 1A). To insert flanking loxP sites, exon 2 of Tβ4 was chosen as the target for conditional deletion because exon 2 encompasses approximately 75% of the coding sequence, includes the start codon, and encodes 33 amino acids of the 43-amino acid Tβ4 protein. Thus, the removal of exon 2 is highly likely to cause a deletion and frameshift, resulting in a null allele. Two different CRISPR gRNAs (gR#1 and gR#2) were designed to separately target the distinct locations of Tβ4 introns 1–2 and 2–3, respectively, producing the floxed Tβ4 allele (Figure S1). To obtain homozygous Tβ4^flox/flox^ mice, hemizygous Tβ4^flox/+^ mice were interbred, and the offspring were genotyped by PCR. The presence of the loxP site was detected in the transgenic mice (Figure 1B). Among these mice, hemizygous Tβ4^flox/+^ mice had wild-type (wt, 195 bp) and mutant (229 bp) forms of the Tβ4 allele, and homozygous Tβ4^flox/flox^ (Tβ4-flox) mice contained only the Tβ4 mutant allele. Homozygous Tβ4^flox/flox^ mice appeared normal, were fertile, and were born at the expected Mendelian ratio.

Because Tβ4 has been shown to be mainly expressed by activated HSCs [16,17], primary HSCs (pHSCs) were isolated from Tβ4-flox mice and cultured for 7 days to facilitate spontaneous activation and check whether Tβ4 could be deleted by the Cre-LoxP system in these cells. On Day 4, pHSCs were treated with adenoviral vectors having Cre recombinase (AdCre) or green fluorescent protein (AdGFP). Compared with freshly isolated pHSCs (Day 0), AdGFP-treated pHSCs had significantly increased levels of Tβ4 on Day 7, as measured by qRT-PCR (Figure 1C). However, the Tβ4 RNA level in pHSCs treated with AdCre was remarkably decreased compared with that in AdGFP-exposed pHSCs. These results validate the efficacy of Tβ4 deletion by using Tβ4-flox mice and suggest that Tβ4-flox mice are available tools to conditionally knock out Tβ4 in vivo.

### 3.2. HSC-Targeted Knockout of Tβ4 Prevents Liver Fibrosis in Mice

After confirming the successful deletion of Tβ4 by the Cre-LoxP system in pHSCs isolated from Tβ4-flox mice, we examined the physiological function of Tβ4 in the liver by generating double-transgenic (DTG) mice in which Tβ4 could be selectively deleted in activated HSCs in vivo. Because α-Sma is a reliable and widely used marker of activated/myofibroblastic HSCs [3,22], α-Sma-Cre-ER^T2^ mice in which tamoxifen (TMX)-inducible Cre activity is influenced by the α-Sma promoter were mated with Tβ4-flox mice, generating DTG mice. The offspring from this mating were genotyped to determine the presence of the Cre transgene and the floxed Tβ4 allele (data not shown). Thereafter, Cre-positive and homozygous Tβ4^flox/flox^ DTG mice were used for the next experiment.

To test the effect of Cre-mediated Tβ4 deletion in vivo and investigate the function of Tβ4 in HSCs, we first used a carbon tetrachloride (CCl_4_)-induced liver fibrosis model (Figure S2A). In the experimental animal model of liver fibrosis, liver morphology was distorted, and the levels of serum alanine aminotransferase (ALT) and aspartate aminotransferase (AST) were significantly elevated in CCl_4_-exposed mice compared with corn oil-treated mice (Figure S2B,C). Distinct liver fibrosis was confirmed by the accumulation of collagen and higher amounts of hepatic hydroxyproline, which is a biochemical measurement quantifying liver fibrosis, and α-Sma in mice treated with CCl_4_ than mice treated with corn oil (Figure S2B–D). Tβ4 was also elevated in the fibrotic group (Figure S2D). After confirming that CCl_4_ induced severe hepatic fibrosis in mice, DTG mice receiving CCl_4_ or corn oil (CON) were intraperitoneally injected with TMX to knockout Tβ4 (Figure 1D). The same volume of vehicle (VEH) with TMX was given to the CON + VEH and CCl_4_ + VEH groups. The hepatic Tβ4 level was significantly elevated in the CCl_4_ + VEH group compared with that in the CON + VEH group, but the level of Tβ4 apparently declined in the CCl_4_ + TMX group, as examined by qRT-PCR and immunoblotting (Figure 1E,F). Immunohistochemistry staining for Tβ4 presented that the hepatic accumulation of Tβ4-positive cells was markedly reduced in the CCl_4_ + TMX group compared with the CCl_4_ + VEH group (Figure 1G). Consistent with our previous findings [16,17], the livers in the CON + VEH group hardly expressed Tβ4 (Figure 1E–G). TMX itself did not influence hepatic Tβ4 expression (data not shown). To examine the selective deficiency of Tβ4 in HSCs, Tβ4 expression was measured in the pHSCs isolated from these mice. As expected, qRT-PCR and immunoblot analyses showed that in pHSCs, the Tβ4 level was significantly higher in the CCl_4_ + VEH group than the CON + VEH group, whereas this level greatly decreased in the CCl_4_ + TMX group, which had enhanced Cre expression compared to the CCl_4_ + VEH group (Figure 1H,I). These data suggest that TMX successfully induced Cre expression, which selectively deleted Tβ4 in HSCs in vivo.

**Figure 2:**
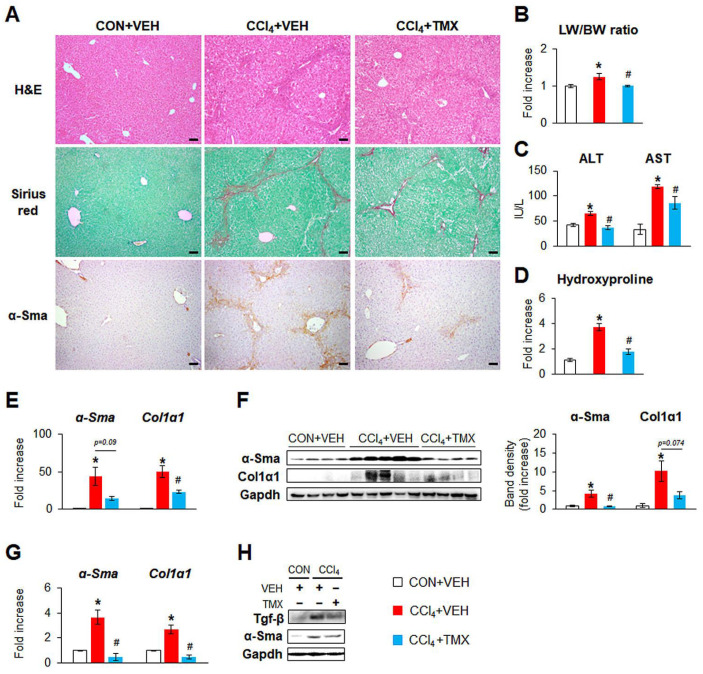
HSC-targeted Tβ4 deletion alleviates CCl_4_-induced liver injury and fibrosis. (**A**) Representative images of hematoxylin and eosin (H&E) staining, Sirius red staining, and immunostaining for α-Sma in liver sections from DTG mice receiving corn oil (CON) + vehicle (VEH), carbon tetrachloride (CCl_4_) + VEH, or CCl_4_ + tamoxifen (TMX) (scale bar = 50 μm). (**B**) Relative ratio of liver weight to body weight (LW/BW), (**C**) serum levels of ALT and AST, and (**D**) hepatic hydroxyproline contents in livers of all mice from each group. (**E**) qRT-PCR analysis of hepatic α-Sma and Col1α1 in representative mice from each group (*n* = 4 per group). (**F**) Immunoblots and cumulative densitometric analysis of hepatic α-Sma and Col1α1 in representative mice from each group, CON + VEH (*n* = 4), CCl_4_ + VEH (*n* = 5), and CCl_4_ + TMX (*n* = 4). Gapdh was used as an internal control. All band densities were normalized versus Gapdh. (**G**) qRT-PCR analysis of α-Sma and Col1α1 in pHSCs isolated from livers of representative mice from each group (*n* = 3 per group). Results of relative expression values are graphed as mean ± s.e.m. (Student’s *t*-test; * *p* < 0.05 vs. CON + VEH group, ^#^ *p* < 0.05 vs. CCl_4_ + VEH group). (**H**) Immunoblots of Tgf-β and α-Sma in these cells. Gapdh was used as an internal control. Immunoblots shown represent one of three experiments with similar results.

We investigated whether HSC-targeted Tβ4 deletion influenced the liver response to CCl_4_-induced damage. H&E staining and Sirius red staining revealed that severe hepatic injury and fibrotic nodules were alleviated in the CCl_4_ + TMX group compared with the CCl_4_ + VEH group (Figure 2A, top and middle panel). The increases in liver weight-to-body weight (LW/BW) ratio, levels of serum ALT/AST, and hydroxyproline contents in the CCl_4_ + VEH group were significantly reduced in the CCl_4_ + TMX group (Figure 2B–D). Immunohistochemistry staining for α-Sma presented that the apparent accumulation of α-Sma-expressing HSCs in the liver in the CCl_4_ + VEH group was markedly mitigated in the CCl_4_ + TMX group (Figure 2A, bottom panel). Fibrotic changes in these mice were confirmed by Western blot analysis, which showed that the significant increase in the HSC activation markers α-Sma and Col1α1 in the CCl_4_ + VEH group compared with the CON + VEH group was significant or tended to be alleviated in the livers of the CCl_4_ + TMX group (Figure 2E,F). In addition, pHSCs from the CCl_4_ + TMX group had significantly lower levels of α-Sma and transforming growth factor beta (Tgf-β) than those in cells from the CCl_4_ + VEH group (Figure 2G,H). TMX itself did not influence liver function, histomorphology, or fibrosis (Figure S3).

**Figure 3:**
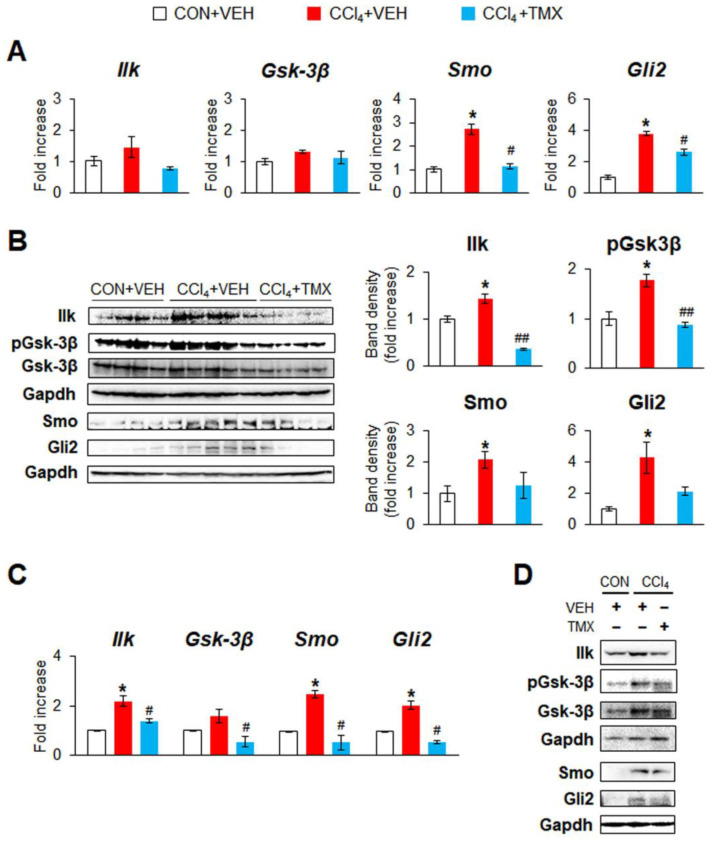
Tβ4 suppression in activated HSCs reduces expression of Hh signaling. (**A**) qRT-PCR analysis of Ilk, Gsk-3β, Smo, and Gli2 in the livers from representative DTG mice receiving corn oil (CON) + vehicle (VEH) (*n* = 3), carbon tetrachloride (CCl_4_) + VEH (*n* = 3), or CCl_4_ + tamoxifen (TMX) (*n* = 4). (**B**) Representative immunoblots and cumulative densitometric analysis of hepatic Ilk, phosphorylated (p) Gsk-3β, Gsk-3β, Smo, and Gli2 in representative mice from each group CON + VEH (*n* = 4), CCl_4_ + VEH (*n* = 5), and CCl_4_ + TMX (*n* = 4). Gapdh was used as an internal control. All band densities were normalized versus Gapdh, except that pGsk-3β was normalized to total Gsk-3β. (**C**) qRT-PCR analysis of Ilk, Gsk-3β, Smo, and Gli2 in pHSCs isolated from livers of representative mice from each group (*n* = 3 per group). Results of relative expression values are graphed as mean ± s.e.m. (Student’s *t*-test; * *p* < 0.05 vs. CON + VEH group, ^#^ *p* < 0.05, ^##^ *p* < 0.005 vs. CCl_4_ + VEH group). (**D**) Immunoblots of Ilk, pGsk-3β, Gsk-3β, Smo, and Gli2 in these cells. Gapdh was used as an internal control. Immunoblots shown represent one of three experiments with similar results.

Given that Tβ4 is associated with Hh signaling in HSC transdifferentiation [16], the activation of Hh signaling was examined in these mice. Although the mRNA levels of Ilk, an activator of Smo, and pGsk-3β, an inactive form of Gsk-3β that degrades Gli2, were rarely changed among the groups, Ilk and pGsk-3β protein levels were significantly higher in the CCl_4_ + VEH group than in the CON + VEH group, and this upregulation led to an increase in Smo and Gli2 at both the mRNA and protein levels in mice with fibrotic livers (Figure 3A,B). Tβ4 knockout, however, abrogated these significant increases. These expression changes in these mice were also verified in pHSCs and showed that pHSCs from the CCl_4_ + TMX group had significantly lower levels of Ilk, pGsk-3β, Smo and Gli2 than those from the CON + VEH group (Figure 3C,D). Taken together, these findings display that HSC-specific Tβ4 deletion ameliorates CCl_4_-induced liver fibrosis and injury by suppressing Hh signaling in mice.

### 3.3. Blocking Tβ4 in HSCs Attenuates Bile Duct Ligation (BDL)-Caused Liver Fibrosis in Mice

**Figure 4:**
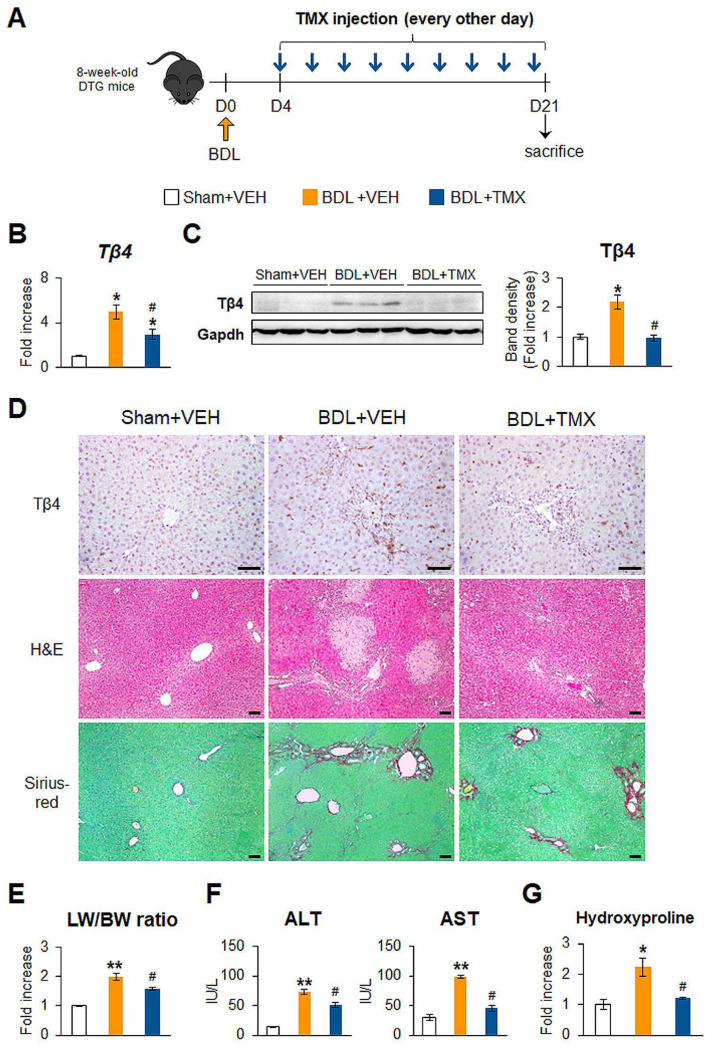
Tβ4 deficiency in HSCs attenuates BDL-induced liver injury and fibrosis. (**A**) Schematic diagram showing the timing of bile duct ligation (BDL) surgery and tamoxifen (TMX) injections into double-transgenic (DTG) mice. At 3 weeks after the surgery, all DTG mice receiving either vehicle (VEH) or TMX were sacrificed for harvesting tissue samples. (**B**) qRT-PCR of hepatic Tβ4 in representative DTG mice receiving sham surgery (Sham) + vehicle (VEH) (*n* = 3), BDL + VEH (*n* = 4), or BDL + TMX (*n* = 4). (**C**) Representative immunoblots and cumulative densitometric analysis of hepatic Tβ4 in representative mice from each group (*n* = 3 per group). Gapdh was used as an internal control. Band densities were normalized versus Gapdh. (**D**) Representative images of immunostaining for Tβ4, hematoxylin and eosin (H&E) staining, and Sirius red staining in liver sections of representative mice from each group (scale bar = 50 μm). (**E**) Relative ratio of liver weight to body weight (LW/BW), (**F**) serum levels of ALT and AST and (**G**) hepatic hydroxyproline contents in all mice. Results were graphed as mean ± s.e.m. (Student’s *t*-test; * *p* < 0.05, ** *p* < 0.005 vs. Sham + VEH group, ^#^ *p* < 0.05 vs. BDL + VEH group).

**Figure 5:**
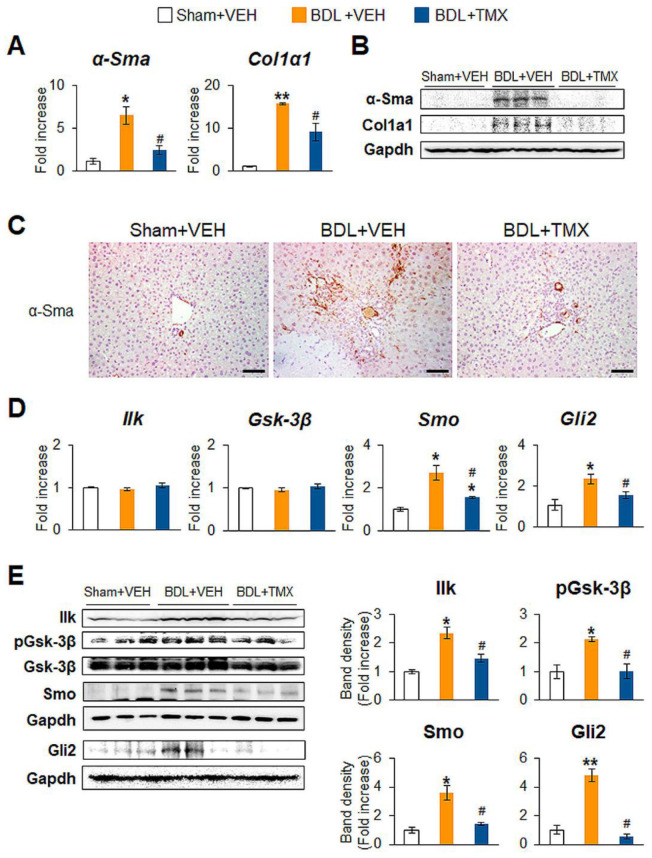
Deleting Tβ4 in HSCs downregulates fibrotic genes and Hh signaling in the liver damaged by BDL. (**A**) qRT-PCR analysis of α-Sma and Col1α1 in liver tissues from representative DTG mice receiving Sham + VEH (*n* = 3), BDL + VEH (*n* = 4), or BDL + TMX (*n* = 4). (**B**) Representative immunoblots of hepatic α-Sma and Col1α1 in mice from representative mice from each group. Gapdh was used as an internal control. (**C**) Representative images of immunostaining for α-Sma in liver sections of from representative mice from each group (scale bar = 50 μm). (**D**) qRT-PCR analysis of hepatic Ilk, Gsk-3β, Smo, and Gli2 in the livers of representative mice from each group, Sham + VEH (*n* = 3), BDL + VEH (*n* = 4), and BDL + TMX (*n* = 4). (**E**) Immunoblots and cumulative densitometric analysis of hepatic Ilk, phosphorylated (p) Gsk-3β, Gsk-3β, Smo, and Gli2 in representative mice from each group (*n* = 3 per group). Gapdh was used as an internal control. All band densities were normalized versus Gapdh, except that pGsk-3β was normalized to total Gsk-3β. Results of relative expression values are graphed as mean ± s.e.m. (Student’s *t*-test; * *p* < 0.05, ** *p* < 0.005 vs. Sham + VEH group, ^#^ *p* < 0.05 vs. BDL + VEH group).

To further validate in vivo roles of Tβ4 in hepatic fibrosis, we employed bile duct ligation (BDL) as an additional animal model of liver fibrosis. CCl_4_ is a chemical that damages hepatocytes and induces inflammation and fibrosis, whereas BDL is a surgical method to induce the proliferation of biliary epithelial cells, followed by inflammation and fibrosis [22,26]. DTG mice underwent sham surgery or BDL for 3 weeks and were injected with VEH or TMX [22] (Figure 4A). As shown in the CCl_4_ model, hepatic Tβ4 expression was greatly elevated in the BDL + VEH group compared with the Sham + VEH group, whereas this level was significantly alleviated in the BDL + TMX group compared with the BDL + VEH group, as measured by qRT-PCR and immunoblotting (Figure 4B,C). Immunostaining for Tβ4 presented that the accumulation of cells positive for Tβ4 in the liver sections from the BDL + TMX group was noticeably decreased in the BDL + TMX group (Figure 4D, top panel). Mice injured by BDL had severe liver damage, such as massive cell death, excessive inflammation, and increases in the LW/BW ratio and ALT/AST level compared with control mice subjected to sham operation (Figure 4D, middle panel, E, F). However, compared with mice in the BDL + VEH group, mice lacking Tβ4 exhibited significantly mild injury, although these mice were subjected to the BDL operation. In addition, hepatic fibrosis caused by BDL was remarkably ameliorated in the BDL + TMX group compared with the BDL + VEH group (Figure 4D bottom panel, G). TMX itself hardly impacted the liver in the sham surgery group (Figure S4). The amounts of α-Sma and Col1α1 were higher in the BDL + VEH group than the Sham + VEH group and were remarkably decreased in the BDL + TMX group (Figure 5A,B). The reduction in HSC activation in the BDL + TMX group was supported by immunostaining for α-Sma, which showed less accumulation of α-Sma-expressing HSCs in the BDL + TMX group than in the BDL + VEH group (Figure 5C). Compared with those in the Sham + VEH group, the protein levels of Ilk and pGsk-3β and the mRNA and protein levels of Smo and Gli2 were significantly enhanced in the BDL + VEH group with upregulated Tβ4, which was similar to the CCl_4_ model. However, this expression noticeably declined in the BDL + TMX group with Tβ4 suppression (Figure 5D,E). These findings demonstrate that conditional disruption of Tβ4 in activated HSCs alleviates BDL-induced liver injury and fibrosis by inhibiting Hh signaling in mice.

### 3.4. Tβ4 Restoration Reverses the Effect of Tβ4 Depletion on Mitigating Liver Fibrosis

**Figure 6:**
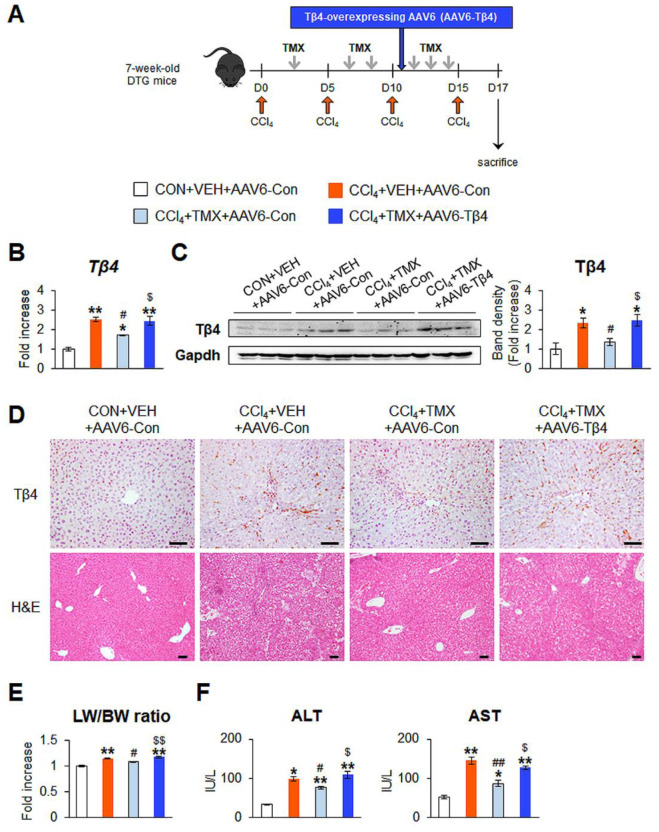
AAV6-mediated re-expression of Tβ4 worsens CCl_4_-induced liver damage in DTG mice treated with tamoxifen. (**A**) Schematic diagram showing the timing of CCl**_4_**, TMX, and Tβ4-expressing adeno-associated virus type-6 (AAV6) administrations into double-transgenic (DTG) mice. At 2 days after the last injection of CCl**_4_**, all DTG mice receiving corn oil (CON) + vehicle (VEH) + AAV6-Con, CCl**_4_** + VEH + AAV6-Con, CCl**_4_** + TMX + AAV6-Con, and CCl**_4_** + TMX + AAV6-Tβ4 were sacrificed. (**B**) qRT-PCR of Tβ4 in the livers from representative DTG mice receiving CON + VEH + AAV6-Con (*n* = 3), CCl**_4_** + VEH + AAV6-Con (*n* = 3), CCl**_4_** + TMX + AAV6-Con (*n* = 4), or CCl**_4_** + TMX + AAV6-Tβ4 (*n* = 4). (**C**) Representative immunoblots and cumulative densitometric analysis of hepatic Tβ4 in representative mice from each group (*n* = 3 per group). Gapdh was used as an internal control. Band densities were normalized versus Gapdh. (**D**) Representative images of immunostaining for Tβ4 and hematoxylin and eosin (H&E) staining in liver sections of representative mice from each group (scale bar = 50 μm). (**E**) Relative ratio of liver weight to body weight (LW/BW); (**F**) serum levels of ALT and AST in all mice from each group. Results were graphed as mean ± s.e.m. (Student’s *t*-test; * *p* < 0.05, ** *p* < 0.005 vs. CON + VEH + AAV6-Con group, ^#^ *p* < 0.05, ^##^ *p* < 0.005 vs. CCl**_4_** + VEH + AAV6-Con group, ^$^ *p* < 0.05, ^$$^ *p* < 0.005 vs. CCl**_4_** + TMX + AAV6-Con group).

To verify the action of Tβ4 in activated HSCs and liver fibrosis in mice, exogenous Tβ4 was administered to CCl_4_-treated DTG mice to restore Tβ4 deficiency in these mice. To conditionally enhance Tβ4 re-expression in the livers of TMX-treated DTG mice, adeno-associated virus type-6 (AAV6), which has been reported to mainly target HSCs [24], was administered. After the 3rd injection of CCl_4_, DTG mice received a single intravenous administration of AAV6-Tβ4 or AAV6-control (AAV6-Con), and then residual TMX and CCl_4_ injections were administered to these mice in an identical regimen (Figure 6A). The time point of AAV6 administration was determined based on previous studies [23,27,28].

**Figure 7:**
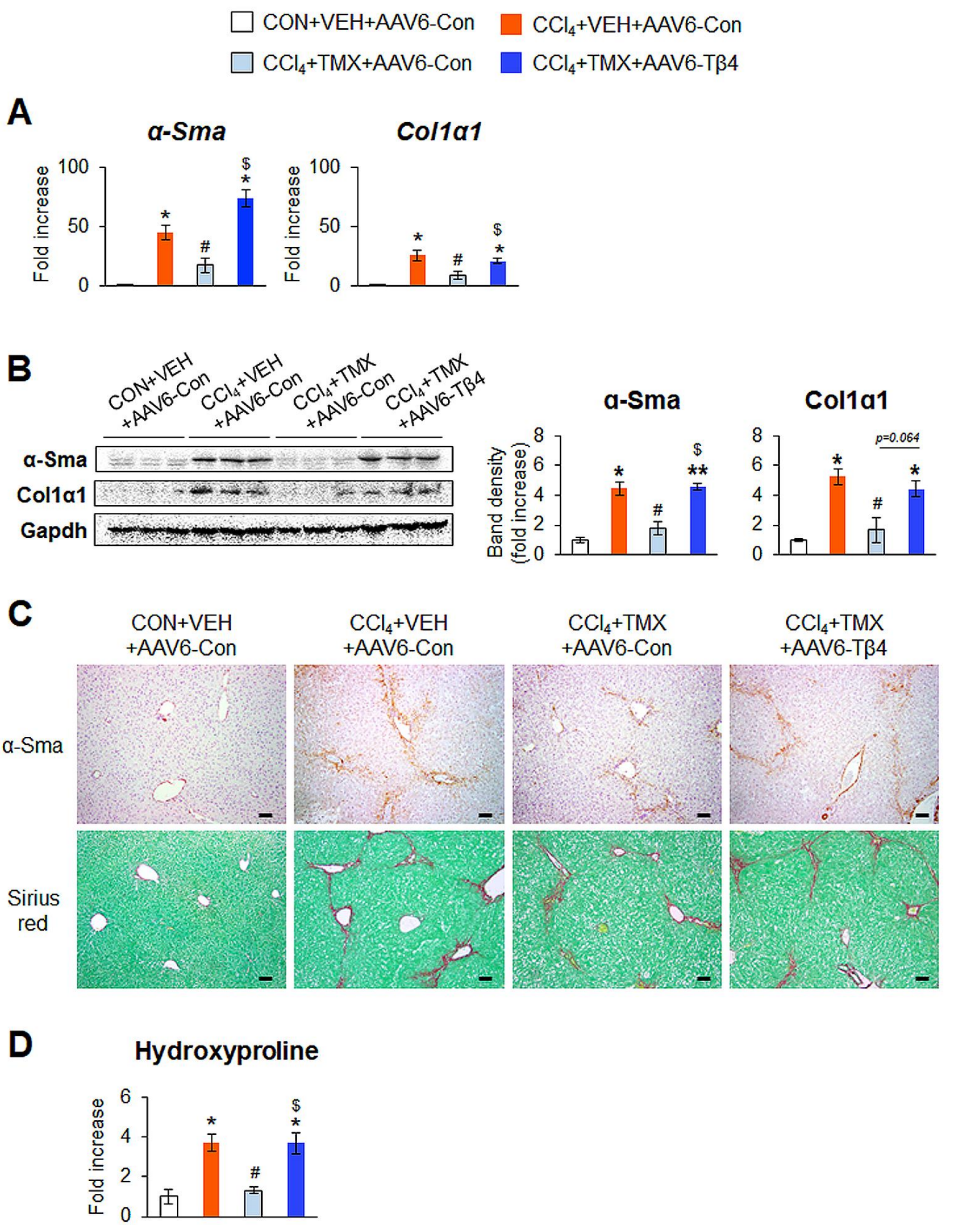
Re-expression of Tβ4 by AAV6-Tβ4 activates HSCs and aggravates liver fibrosis in tamoxifen-treated DTG mice. (**A**) qRT-PCR analysis of hepatic α-Sma and Col1α1 in representative DTG mice receiving CON + VEH + AAV6-Con (*n* = 3), CCl_4_ + VEH + AAV6-Con (*n* = 3), CCl_4_ + TMX + AAV6-Con (*n* = 4), or CCl_4_ + TMX + AAV6-Tβ4 (*n* = 3). (**B**) Representative immunoblots and cumulative densitometric analysis of hepatic α-Sma and Col1α1 in representative mice from each group (*n* = 3 per group). Gapdh was used as an internal control. Band densities were normalized versus Gapdh. (**C**) Representative images of immunostaining for α-Sma and Sirius red staining in liver sections from each group (scale bar = 50 μm). (**D**) Hepatic hydroxyproline contents in livers of all mice from each group. Results were graphed as mean ± s.e.m. (Student’s *t*-test; * *p* < 0.05, ** *p* < 0.005 vs. CON + VEH + AAV6-Con group, ^#^ *p* < 0.05 vs. CCl_4_ + VEH + AAV6-Con group, ^$^ *p* < 0.05 vs. CCl_4_ + TMX + AAV6-Con group).

**Figure 8:**
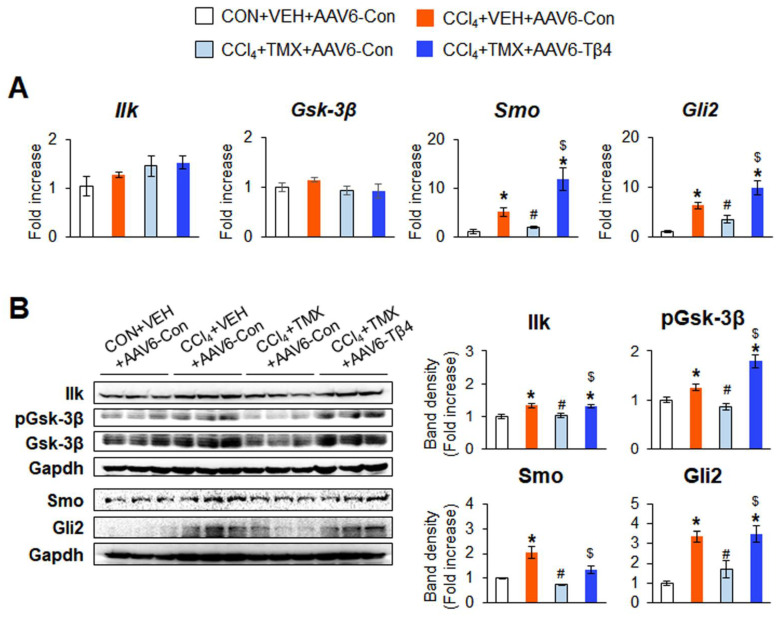
Re-expression of Tβ4 by AAV6 provokes activation of Hh signaling in tamoxifen-treated DTG mice. (**A**) qRT-PCR analysis of Ilk, Gsk-3β, Smo, and Gli2 in the liver tissues from representative DTG mice receiving CON + VEH + AAV6-Con (*n* = 3), CCl_4_ + VEH + AAV6-Con (*n* = 3), CCl_4_ + TMX + AAV6-Con (*n* = 4), and CCl_4_ + TMX + AAV6-Tβ4 (*n* = 3). Results of relative expression values are graphed as mean ± s.e.m. (**B**) Representative immunoblots and cumulative densitometric analysis of hepatic Ilk, phosphorylated (p) Gsk-3β, Gsk-3β, Smo, and Gli2 in representative mice from each group (*n* = 3 per group). Gapdh was used as an internal control. All band densities were normalized versus Gapdh, except that pGsk-3β was normalized to total Gsk-3β. Results of relative expression values are graphed as mean ± s.e.m. (Student’s *t*-test; * *p* < 0.05 vs. CON + VEH + AAV6-Con group, ^#^ *p* < 0.05 vs. CCl_4_ + VEH + AAV6-Con group, ^$^ *p* < 0.05 vs. CCl_4_ + TMX + AAV6-Con).

AAV6-Tβ4 administration upregulated hepatic Tβ4 expression in the CCl_4_ + TMX + AAV6-Tβ4 group, restoring it to a level similar to that in the CCl_4_ + VEH + AAV6-Con group, as measured by qRT-PCR and immunoblot analysis (Figure 6B,C). The recovered Tβ4 level was supported by immunohistochemistry staining for Tβ4, which showed that Tβ4-expressing cells were noticeably increased in the livers in the CCl_4_ + TMX + AAV6-Tβ4 group compared with the CCl_4_ + TMX + AAV6-Con group (Figure 6D, top panel). To identify whether AAV6-mediated restoration of Tβ4 exacerbated liver damage in the context of fibrosis, we checked the liver morphology and degree of liver fibrosis in these mice. The improvements in liver morphology and function due to Tβ4 suppression were worsened by Tβ4 recovery in the CCl_4_ + TMX + AAV6-Tβ4 group (Figure 6D, bottom panel, E,F). The amounts of α-Sma and Col1α1 were significantly elevated in the CCl_4_ + TMX + AAV6-Tβ4 group compared with the CCl_4_ + TMX + AAV6-Con group, and these levels in the CCl_4_ + TMX + AAV6-Tβ4 group were similar to those in the CCl_4_ + VEH + AAV6-Con group (Figure 7A,B). The accumulation of α-Sma-expressing cells and fibrotic nodules was more evident in the CCl_4_ + TMX + AAV6-Tβ4 group than in the CCl_4_ + TMX + AAV6-Con group (Figure 7C). Hepatic hydroxyproline levels confirmed that the CCl_4_ + TMX + AAV6-Tβ4 group had markedly more liver fibrosis than the CCl_4_ + TMX + AAV6-Con group (Figure 7D). Consistent with the re-establishment of Tβ4 expression and liver fibrosis, activated Hh signaling was also observed. The levels of Ilk, pGsk-3β, Smo, and Gli2 were enhanced in the CCl_4_ + TMX + AAV6-Tβ4 group compared with the CCl_4_ + TMX + AAV6-Con group, as evaluated by qRT-PCR and immunoblotting (Figure 8A,B). These findings reveal that the restoration of Tβ4 in Tβ4-deficient HSCs activates Hh signaling and promotes liver fibrosis, indicating that Tβ4 is one of the main regulators of HSC activation and liver fibrosis.

## 4. Discussion

Since HSCs are key modulators in the development of liver fibrosis, HSCs have been major targets for antifibrotic therapies [1,29]. Inhibiting HSC activation is considered the key strategy to reduce the number of ECM-producing cells and can prevent and treat liver fibrosis [2,29]. Thus, to unveil the functional effect of Tβ4 on HSC transdifferentiation in the liver, we used the two most widely used animal models of liver fibrosis: the CCl_4_ model and the BDL model. α-Sma is the most well-known marker of activated/myofibroblastic HSCs, and these cells expressing α-Sma proliferate and accumulate in the CCl_4_ or BDL model [22,23]. Based on our previous studies that showed that α-Sma-positive HSCs express Tβ4 in fibrotic livers [16,17], we used the TMX-inducible Cre/loxP system to conditionally knock out Tβ4 in α-Sma-expressing cells. In both the CCl_4_ and BDL models, TMX injection significantly downregulated Tβ4 expression compared to that in the vehicle-injected group (Figure 1 and Figure 4). Moreover, HSC-targeted Tβ4 deletion reduced the level of α-Sma and the deposition of fibrillar collagen, leading to a reduction in liver fibrosis (Figure 2, Figure 4 and Figure 5). These findings suggest that deleting Tβ4 in activated HSCs could be an effective strategy to suppress HSC activation and eventually reduce liver fibrosis.

Although two different experimental models of liver fibrosis were used to examine the in vivo function of Tβ4, the relatively short-term injection of CCl_4_ (four CCl_4_ injections for 20 days) compared with the classical CCl_4_ injury model (CCl_4_ injection for 6~10 weeks) is a limitation of the research. However, because long-term exposure to TMX itself is reported to be toxic to the liver and stomach [30,31,32], we had to adopt the short-term injection model of CCl_4_ based on the TMX exposure period, which was verified in another study using the TMX-inducible Cre-loxP model system [23]. We confirmed severe hepatic damage including fibrosis in mice receiving a short-term injection of CCl_4_, and we confirmed that TMX itself hardly influenced our experimental system except for Cre activation (Figures S2–S4). The successful deletion of Tβ4 in HSCs and no harmful effects of Tβ4 on the uninjured liver support that the animal experimental design is appropriate. Nevertheless, further research is required to examine the long-term influences of Tβ4 on the progression of chronic liver fibrosis and cirrhosis to validate relevance of Tβ4 in a chronic model more closely reflecting human liver fibrosis.

Tβ4 was shown to regulate HSC activation through Hh signaling, an essential controller of HSC activation [16]. In particular, the Smo–Gli2 axis, an activator of Hh signaling, is crucial in orchestrating liver fibrosis [22,33,34,35]. During HSC activation, Tβ4 promoted the activation of the Smo–Gli2 axis by modulating the activity of ILK and pGSK-3β and directly interacting with Smo and Gli2 [16]. In line with these findings, an in vivo study using mice lacking Tβ4 in activated HSCs demonstrated that Tβ4 depletion in HSCs impaired the Hh activation. Although Tβ4 deletion downregulated only the protein but not the mRNA levels of ILK and pGSK-3β in whole liver tissues in the CCl_4_ and BDL models (Figure 3 and Figure 5), the data obtained from the pHSC experiments clearly showed that TMX-inducible Tβ4 deletion significantly reduced the mRNA levels of ILK and GSK-3β in these cells (Figure 3). In addition, given that Tβ4 is known to mainly regulate the stability of the ILK protein and the phosphorylation of GSK-3β [16,36,37,38], our data clearly suggest that Tβ4 regulates ILK and GSK-3β.

Tβ4 was initially recognized as a G-actin-binding protein and is capable of regulating cytoskeleton reorganization and cell motility [4,5,9]. Recently, novel functions of Tβ4 have been revealed based on its ability to impact various biological activities, such as cell survival, migration, repair, and angiogenesis [4,10,11,39]. However, most findings have been obtained from investigating the function of exogenous or synthetic Tβ4 that was applied extracellularly or in a paracrine manner [4,5,9]. A relatively small number of studies have paid attention to the in vivo functions of endogenous Tβ4. There have been several reports published studying the role of endogenous Tβ4 in heart and hair growth in Tβ4 transgenic mice, including ubiquitous knockout, tissue-specific knockout, or overexpression mice [40,41,42,43]. Our previous study showed that mice overexpressing Tβ4 were more susceptible to the development of liver fibrosis than WT mice [16]. The present study also demonstrates that HSC-specific deletion of Tβ4 prevents fibrosis in the liver, and this effect is reversed by the re-expression of Tβ4 in HSCs, confirming that endogenous Tβ4 expressed by activated HSCs has an in vivo function in facilitating HSC activation and liver fibrosis. The reason why our finding on the endogenous action of hepatic Tβ4 opposes the beneficial actions of exogenous Tβ4 shown by other studies remains unknown. However, it is highly possible that there is a difference between the effects of endogenous and exogenous Tβ4 [5]. Banerjee et al. reported that whole-body or cardiac-specific knockout of Tβ4 did not influence overall embryonic development, heart development, or adult cardiac function [42]. These data are also contrary to many other findings showing that exogenous Tβ4 administration improves cardiovascular function and repair [38,44,45,46]. Notably, in cancer studies, many reports have shown that Tβ4 is upregulated in breast, colorectal, and pancreatic cancer and is associated with poor prognosis [47,48,49,50,51]. Therefore, in advance of the therapeutic use of Tβ4, further studies and evidence are needed to determine the roles of endogenous Tβ4 and should discuss the potential use of exogenous or synthetic Tβ4 regarding the action of endogenous Tβ4.

In conclusion, our results show that HSC-targeted Tβ4 deletion reduces liver fibrosis in vivo by suppressing Hh signaling and that Tβ4 regulates HSC activation during liver fibrosis, suggesting that Tβ4 could be a good target for controlling HSC activation in the development of effective therapeutics for chronic liver disease. In addition, the Tβ4-flox mice that we generated might be a useful tool for investigating the unrevealed functions of endogenous Tβ4 in a tissue- and/or cell-specific manner and contribute to the safe therapeutic application of Tβ4 to the clinic.

## Data Availability

All data were included in this paper.

## Supplementary Materials

The following supporting information can be downloaded at: https://www.mdpi.com/article/10.3390/cells12121658/s1, Figure S1: Design of guide RNAs for CRISPR/Cas9 genome editing; Figure S2: Mice receiving four injections of CCl4 have liver damage with fibrosis and increased level of Tβ4 and Hh signaling; Figure S3: Tamoxifen rarely impacts the liver treated with corn-oil; Figure S4: Tamoxifen hardly influences the liver subjected to sham surgery; Table S1: Primer sequences used for real-time PCR.
