# Supplementary material for: Targeted Deletion of Thymosin Beta 4 in Hepatic Stellate Cells Ameliorates Liver Fibrosis in a Transgenic Mouse Model
Source: Cells. 2023 Jun 18;12(12):1658. doi: 10.3390/cells12121658 (PMC10297343; doi:10.3390/cells12121658)
Supplement: Supplementary file 1 [file cells-12-01658-s001.zip › cells-2397443-supplementary.pdf]

Supplementary materials

Supplementary Table S1. Primer sequences used for real-time PCR

**Gene**	**Forward**	**Reverse**
Tβ4	ATGTCTGACAAACCCGATATGGC	CCAGCTTGCTTCTCTTGTTCA
α-Sma	AAACAGGAATACGACGAAG	CAGGAATGATTTGGAAAGGA
Col1α1	GAGCGGAGAGTACTGGATCG	GCTTCTTTTCCTTGGGGTTC
Ilk	GTGAATGAGCACGGCAATGTG	CCCATTTTCTCTGCCCGTTCT
Gsk-3β	CAGGGCACCAGAGTTGATCTT	GCTCCCTTGTTGGTGTTCCTA
Smo	CAGCAAGATCTTCGAGACCA	AAGTGGCAGATGAAGGTGAT
Gli2	CAAGCAGAACAGCGAGTCAG	CCTCAGCCTCAGTCTTGACC
S9	GACTCCGGAACAAACGTGAGGT	CTTCATCTTGCCCTGGTCCA

These primer sequences were used for qRT-PCR. The expression level of mRNA was normalized by the expression level of S9 mRNA.

Supplementary Figures

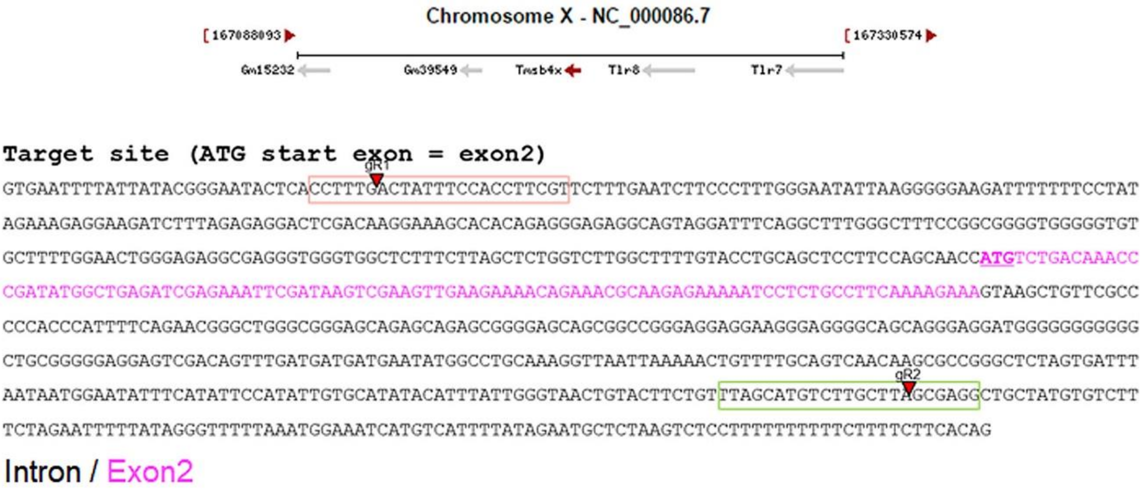

	gRNA sequence	Direction
intron1-2_gR#1	ACGAAGGTGGAAATAGTCAAAGG	-
intron2-3_gR#2	TTAGCATGTCTTGCTTAGCGAGG	+

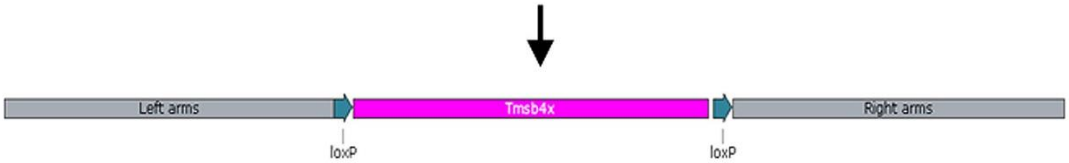

Supplementary Figure S1. Design of guide RNAs for CRISPR/Cas9 genome editing. Two different CRISPR gRNAs (gR#1, gR#2) were designed to separately target at the distinct locations of the Tβ4 intron 1-2 and intron 2-3, respectively, producing the floxed Tβ4 allele. Red inverted triangle indicates the insertion site of gR#1 and gR#2, respectively.

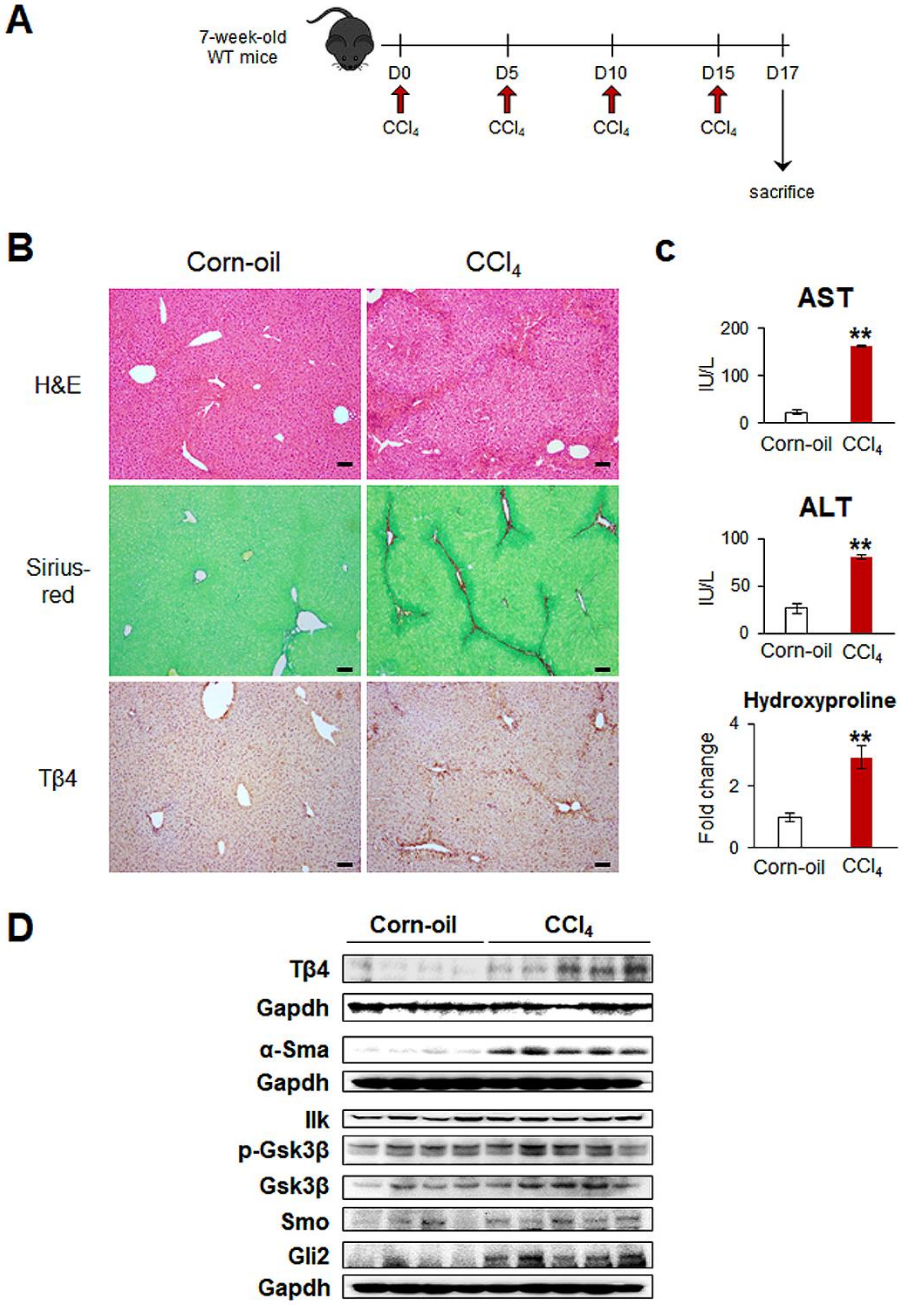

Supplementary Figure S2. Mice receiving four injections of CCl_4 have liver damage with fibrosis and increased level of Tβ4 and Hh siganling

(A) Schematic diagram showing the timing of four injections of carbon tetrachloride (CCl_4) into wild-type (WT) mice. At 2 days after the last injection of CCl_4, all WT mice receiving either CCl_4 (n=5) or corn-oil (n=4) were sacrificed. (B) Representative images of hematoxylin and eosin (H&E) staining, Sirius red staining and immunostaining for Tβ4 in liver sections of representative

mice from each group (scale bar = 50 μm). (C) Serum levels of AST and ALT, and hepatic hydroxyproline contents in the livers of all mice from each group (student's t-test; $^{**}p<0.005$ vs. Corn-oil). (D) Representative immunoblots of Tβ4, α-Sma, Ilk, pGsk-3β, Gsk-3β, Smo, and Gli2 in the livers from each group. Gapdh was used as an internal control.

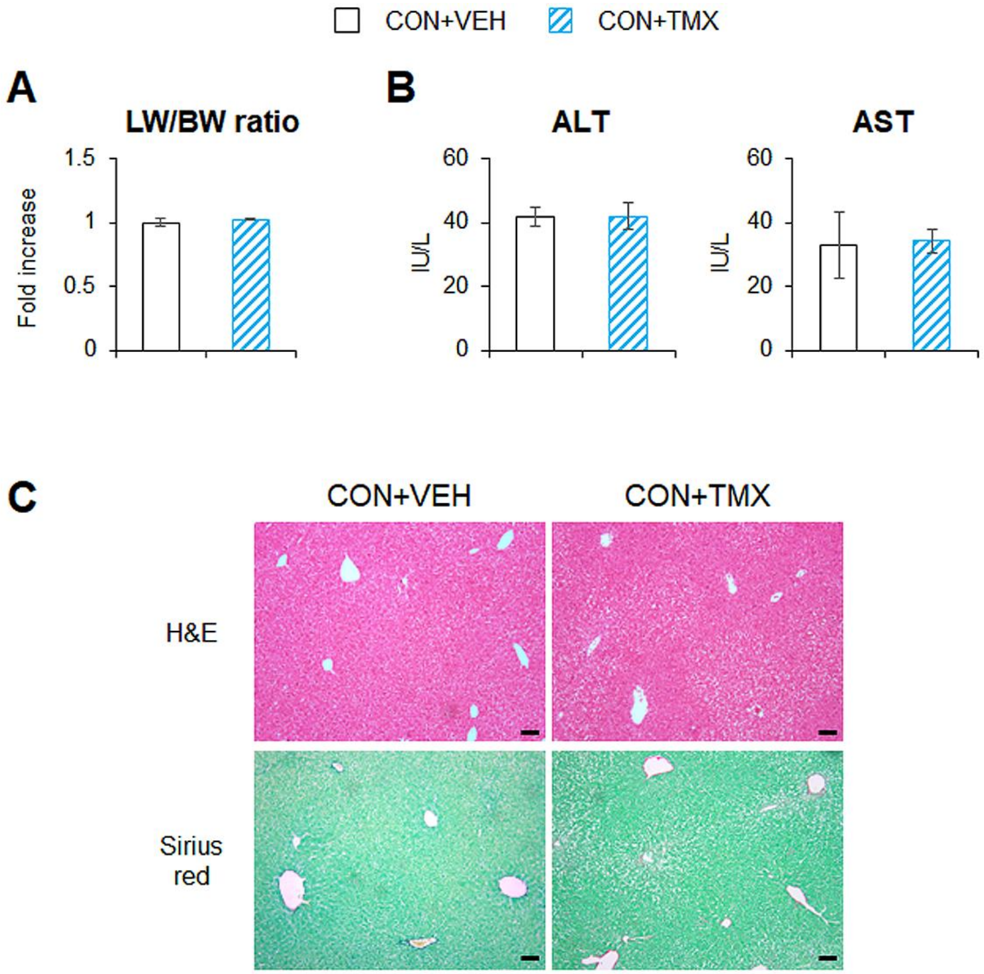

Supplementary Figure S3. Tamoxifen rarely impacts the liver treated with corn-oil. (A) Relative ratio of liver weight to body weight (LW/BW) and (B) serum levels of ALT and AST in corn-oil injected DTG mice treated with either vehicle (CON+VEH) or tamoxifen (CON+TMX) (n=4 per group). Results of relative expression values are graphed as mean±s.e.m. (C) Representative images of hematoxylin and eosin (H&E) staining and Sirius red staining in liver sections from representative mice from each group (scale bar = 50 μm).

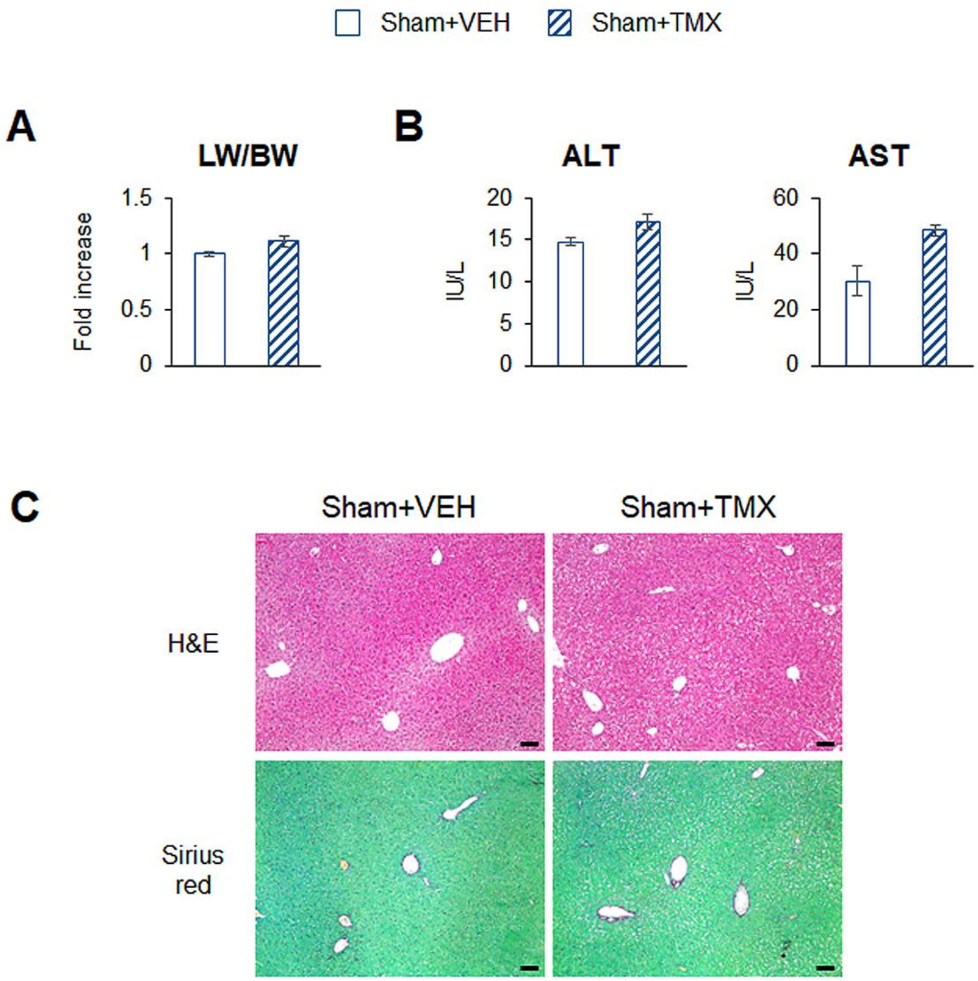

Supplementary Figure S4. Tamoxifen hardly influences the liver subjected to sham surgery. (A) Relative ratio of liver weight to body weight (LW/BW) and (B) serum levels of ALT and AST in DTG mice subjected to sham surgery and treated with either vehicle (Sham+VEH) or tamoxifen (Sham+TMX) (n=4 per group). Results of relative expression values are graphed as mean±s.e.m. (C) Representative images of hematoxylin and eosin (H&E) staining and sirius red staining in liver sections from representative mice from each group (scale bar = 50 μm).
